# Restrictive *versus* liberal oxygenation in patients undergoing cardiopulmonary bypass-assisted heart surgery: a randomised controlled trial

**DOI:** 10.1016/j.bja.2025.08.005

**Published:** 2025-08-19

**Authors:** Sebastian Wiberg, Christian H. Møller, Jesper Kjaergaard, Astrid D. Mikkelsen, Hasse-Møller Sørensen, Joakim B. Kunkel, Peter S. Olsen, Dan E. Høfsten, Jesper Ravn, Hanne Ravn, Søren Boesgaard, Christian Hassager, Lars Køber, Jens C. Nilsson

**Affiliations:** 1 Department of Cardiothoracic Anesthesiology and Intensive Care, Copenhagen University Hospital Rigshospitalet, Copenhagen, Denmark; 2 Department of Cardiology, Copenhagen University Hospital Rigshospitalet, Copenhagen, Denmark; 3 Department of Clinical Medicine, Faculty of Health Sciences, University of Copenhagen, Copenhagen, Denmark; 4 Department of Cardiothoracic Surgery, Copenhagen University Hospital Rigshospitalet, Copenhagen, Denmark; 5 Department of Anesthesiology and Intensive Care, Odense University Hospital, Odense, Denmark

**Keywords:** aortic valve replacement, cardiac surgery, cardiopulmonary bypass, coronary artery bypass grafting, hyperoxia, oxygenation

## Abstract

**Background:**

Maintaining adequate oxygen delivery during cardiopulmonary bypass (CPB)-assisted cardiac surgery is crucial, but hyperoxia has been suggested to cause organ injury. We compared the effects of restrictive *vs* liberal oxygenation during CPB and weaning from CPB on clinical outcomes in cardiac surgery.

**Methods:**

We conducted a single-centre, patient- and assessor-blinded randomised trial on adults undergoing CPB-assisted coronary artery bypass grafting, aortic valve replacement, or both. Participants were randomly assigned (1:1) to restrictive (Fio_2_=50%) or liberal (Fio_2_=100%) oxygen therapy during and for the first hour after weaning from CPB. The primary composite outcome was the time to death, stroke, renal failure requiring dialysis, or new-onset or worsening heart failure during follow-up.

**Results:**

Among 1389 participants (mean age, 67 yr [range, 29–85 yr]; 17% female), randomisation to receive Fio_2_ 50% resulted in median Pao_2_ levels of 19–23 kPa during CPB, compared with >60 kPa in participants receiving Fio_2_ 100%. During a median follow-up period of 5.9 yr (interquartile range, 2.5–8.3), 167/695 (24%) participants in the restrictive oxygenation group and 168/694 (24%) participants in the liberal oxygenation group met the primary endpoint (hazard ratio, 1.01 [95% confidence interval, 0.8–1.3]; *P*=0.92). There was no difference in adverse event rates between restrictive and liberal oxygen therapy.

**Conclusions:**

Among patients undergoing elective or urgent CPB-assisted coronary artery bypass grafting, aortic valve replacement, or both, no significant differences were observed in mortality, dialysis-dependent renal failure, stroke, or new-onset or worsening heart failure between a restrictive oxygenation strategy (Fio_2_ 50%) and a liberal oxygenation strategy (Fio_2_ 100%) during CPB and the subsequent weaning period.

**Clinical trial registration:**

NCT02673931

**Editor’s key points**

• Maintaining adequate oxygen delivery during cardiopulmonary bypass-assisted cardiac surgery is crucial, but hyperoxia might exacerbate organ injury.
• This single-centre trial randomised 1389 participants to receive either restrictive (Fio_2_ 50%) or liberal (Fio_2_ 100%) oxygenation during cardiopulmonary bypass for coronary artery bypass grafting, aortic valve replacement, or both.
• The primary composite endpoint comprised death, stroke, new requirement for renal dialysis, and new-onset or worsening heart failure.
• No difference was found between restrictive and liberal oxygen therapy for the composite primary endpoint.
• Oxygenation levels during cardiopulmonary bypass do not impact on organ dysfunction.

Cardiac surgery requiring cardiopulmonary bypass (CPB) is associated with a significant risk of organ injury leading to prolonged length of hospital admission, irreversible organ dysfunction, and death. The risk of major cardiovascular events within 1 yr from surgery is ∼6–7%, whereas the risk of death is reported to be ∼3–4%.^1^^,^^2^ In patients older than 70 yr, the 30-day major cardiovascular event rates are reported to be ∼10%.^3^

The aetiology of organ injury after CPB is complex but likely encompasses multiple overlapping pathways including cellular damage caused by oxidative stress and ischaemia-reperfusion injury resulting in diminished end-organ perfusion. In addition, activation of inflammatory cascades and haemolysis induced by mechanical stress, exposure of blood to foreign surfaces in the CPB circuit, and blood–gas interfaces in the oxygenator may be involved.^4^^,^^5^ During CPB, a key goal is to ensure adequate end-organ oxygen delivery. Accordingly, high Fio_2_ levels have often been used during CPB and during weaning to protect against hypoxia and tissue ischaemia. Furthermore, hyperoxia has been suggested to reduce the prevalence of surgical site infections after major surgery.^6^ Despite perceived beneficial effects, hyperoxia has also been suggested to cause organ injury owing to increased production of reactive oxygen species, enhancement of ischaemia-reperfusion injury, and cardiovascular dysfunction.^7^, ^8^, ^9^, ^10^, ^11^ A recent systematic review concluded that the current evidence base for hyper- *vs* normoxia is small with a risk of bias, and that clinical equipoise exists for further trials to be undertaken.^12^ Current guidelines do not provide specific suggestions for oxygenation targets during CPB.^13^, ^14^, ^15^, ^16^

The present study aimed to investigate the effects of two oxygenation levels in patients undergoing CPB-assisted cardiac surgery. We hypothesised that more restrictive oxygenation (Fio_2_=50%) compared with liberal oxygenation (Fio_2_=100%) during CPB, and for the first hour after weaning from CPB, would reduce the mortality and morbidity from heart, brain, and kidney injury, without increasing the risk of surgical site infection.^17^

## Methods

### Study design

In the GLORIOUS trial, an investigator-initiated, 2×2 factorial design randomised clinical trial, we assigned participants undergoing elective or urgent CPB-assisted coronary artery bypass grafting (CABG), surgical aortic valve replacement (AVR), or both to perioperative treatment with the GLP-1 analogue exenatide *vs* placebo and to undergo restrictive (Fio_2_ 50%) *vs* liberal oxygenation (Fio_2_ 100%) during CPB and the first hour after weaning from CPB. The two interventions were *a priori* and assumed to be independent from each other; here, we report the results of the oxygen intervention. The study design article has been published previously,^17^ and the protocol was made available on clinicaltrials.gov (NCT02673931) before inclusion of the first participant. The protocol was approved by the Danish Medicines Agency (protocol no. HJE-PHARMA-001, EudraCT no. 2015-003050-41) and the Regional Ethics Committee of the Capital Region of Denmark (‘Videnskabsetisk komité C, Region Hovedstaden’, no. H-15010562) before trial initiation in accordance with Danish Legislation.

### Inclusion and exclusion criteria

We included adult participants (≥18 yr of age) undergoing elective or urgent CABG, AVR, or both irrespective of other concomitant valve surgery. A comprehensive list of eligibility criteria can be found in the trial protocol,^17^ and in Supplementary material 1.

### Randomisation

Participants were randomised in a tertiary heart centre in Denmark via an internet-based randomisation algorithm (Zenodotus, Zenodotus v. Dan Eik Høfsten, Hundested, Denmark) using permuted blocks of 4, 8, or 12. The randomisation was stratified by surgical procedure (AVR or not).

### Intervention

The allocated oxygenation strategy was disclosed to the anaesthetist and perfusionist after participants were anaesthetised. The intervention period was defined as the period on CPB and for the first hour after weaning from CPB or until transfer of participants from the operating table to a hospital bed, whichever came first. Participants received a restrictive oxygenation strategy defined as a Fio_2_ of 50% or a liberal oxygenation strategy defined as a Fio_2_ of 100% during the intervention period. The assigned Fio_2_ was administered via the CPB and via the ventilator during and after weaning. The lungs were not ventilated during CPB. During weaning, tidal volume and airway pressures were adjusted in accordance with departmental guidelines. The following safety measure was predefined. If the arterial oxygen saturation (Sao_2_) was below 92% for more than 30 s, or if deemed necessary to ensure safety, the attending physician was allowed to temporarily increase Fio_2_ until the Sao_2_ was equal to or above 92%. All participants, trial investigators, and outcome assessors were blinded for the trial intervention allocation, and the allocation was not disclosed outside the operating room.

### Treatment protocol

Participants received anaesthesia in accordance with contemporary guidelines,^16^ with fentanyl, propofol, and rocuronium used for induction of anaesthesia followed by sevoflurane and remifentanil infusion. A central venous catheter was placed after induction of anaesthesia. Invasive arterial blood pressure was monitored via the radial artery. Transesophageal echocardiography and pulmonary artery catheters were used at the discretion of the anaesthetist, surgeon, or both. During CPB, a fixed pump of 2.4 L min^−1^ m^−2^ body surface area plus 10–20% was applied. The following targets are applied during CPB: partial pressure of arterial CO_2_ from 4.5 to 6.0 kPa, haematocrit above or equal to 21%, and core temperature above or equal to 36.5°C. The patient received standard postoperative care.

### Measurement of oxygen levels

In order to measure the impact of the intervention on patients’ oxygen levels, partial pressures of arterial oxygen (Pao_2_) levels were measured. All Pao_2_ measurements were recorded and stored in a blinded fashion until after unblinding. Before initiation of CPB (i.e. before initiation of the intervention), an arterial blood gas was drawn from the arterial cannula and analysed. During CPB, Pao_2_ levels were automatically recorded every minute using the Spectrum Viper M4 monitor® (Spectrum Medical, Cheltenham, England). For each participant, these Pao_2_ recordings were averaged in 10-min intervals from initiation of CPB to 180 min after initiation of CPB. Per protocol, an arterial blood gas was drawn 10 min after conclusion of CPB and Pao_2_ levels were analysed.

### Primary endpoint

The composite primary endpoint was adjudicated by a committee blinded to treatment allocation. The main outcome was time to the first occurring composite primary endpoint during the trial follow-up period. The composite primary endpoint included:

a. Death from any cause during follow-up.
b. Renal failure requiring any type of renal replacement therapy during follow-up.
c. Stroke during follow-up. Stroke was defined as any sign or symptom of neurological dysfunction persisting for more than 24 h, determined by the treating physician based on clinical information such as CT scan, etc.
d. New-onset or worsening heart failure at any point during follow-up. Heart failure was *a priori* defined as need for mechanical circulatory support at the ICU, inability to close the sternum because of hemodynamic instability, need for inotropic hemodynamic support more than 48 h after initiation of the index surgical procedure after randomisation or a combination of these. In addition, any admission for heart failure during follow-up after discharge from the index admission.

### Secondary endpoints

Secondary endpoints included time in days to occurrence of the individual components of the composite primary endpoint (death, renal failure, stroke, and new-onset or worsening heart failure). Furthermore, secondary endpoints included the incidence of a number of predefined safety endpoints including surgical site infection requiring antibiotics for more than 48 h (excluding routine antibiotic use), acute kidney injury (Kidney Disease: Improving Global Outcomes [KDIGO] stage 2 or higher),^18^ hypoglycaemia (blood glucose <3 mmol L^−1^) or pancreatitis (S-amylase more than three times upper normal limit) during index admission, a relative reduction of LVEF of 50% compared with baseline during index admission, reoperation for bleeding and any cause during index admission, post-surgery myocardial infarction (type 5) during index admission,^19^ and re-admission for cardiovascular causes within 12 months.^17^

### Statistical analyses

All analyses were conducted on the modified intention-to-treat population (i.e. all randomised patients minus patients withdrawing consent before surgery). Throughout, continuous variables are presented as mean (sd) if normally distributed or otherwise as median (interquartile range [IQR]). Categorical variables are presented as numbers (percentages). For the primary endpoint analysis (time to first co-primary endpoint), Kaplan–Meier curves are displayed and Cox proportional hazard models are applied for calculation of hazard ratios (HRs) with 95% confidence intervals (95% CIs). A multivariable Cox proportional hazard model was applied with adjustment for age, sex, body mass index, year of inclusion, procedure, known alcohol or drug use disorder, known heart failure, known dialysis, known pulmonary disease, known diabetes mellitus, previous stroke, previous myocardial infarction, previous percutaneous coronary intervention (PCI), previous CABG, previous AVR, and length of CPB. Covariates in the multivariable model were selected *a priori*. Proportional hazard assumptions were assessed by visual inspection of graphs and the supremum test for proportional hazards assumptions. Analyses of time to renal failure, stroke, and heart failure took competing risk of death into account by censoring at time of death. Rates of predefined adverse events through follow-up between groups were compared with the χ^2^ test or Fisher’s exact test, as appropriate. As a predefined sensitivity analysis, the primary analyses were repeated with the follow-up time limited to 180 days (Supplementary material). As a *post hoc* sensitivity analysis, we tested the effect of the interaction ‘treatment allocation’ by ‘EuroSCORE II above *vs* below median’ on the primary outcome.^20^ A two-sided alpha level of 0.05 was applied throughout. SAS Enterprise guide© version 8.2.5 was used for all analyses (SAS, SAS Institute, Cary, NC, USA).

### Sample size

Interaction between the two trial interventions was not expected, and accordingly, the sample size estimation did not account for effect modification. At a two-sided alpha level of 0.05 and a power of 0.8, the trial would be able to show a 25% relative reduction in the primary endpoint if 1400 patients were included with an event rate of 23% (i.e. 323 events). Accordingly, the trial would be able to show an absolute risk reduction of 5.7%, corresponding to a number-needed-to-treat of 20. The trial was designed as event driven, with inclusion of 1400 patients being followed for a minimum of a year until a total of 323 events had been reached. The expected event rate was based on data from the surgical register at the institution.

## Results

### Participant characteristics

**Fig 1:**
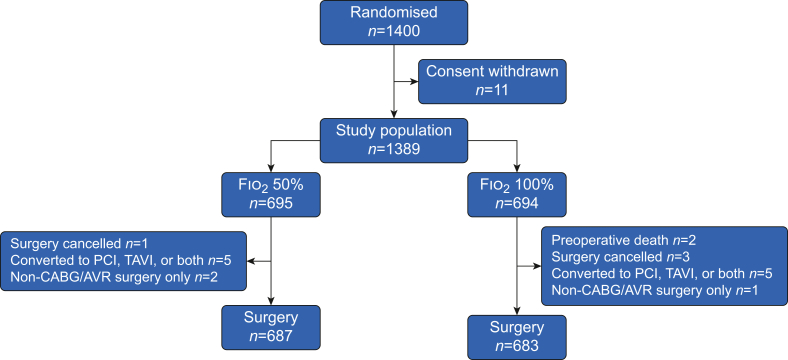
Consort diagram. CABG, coronary artery bypass grafting; PCI, percutaneous coronary intervention; SAVR, surgical aortic valve replacement; TAVI, transcatheter aortic valve implantation.

Participants were included between February 5, 2016, and December 14, 2021, and final follow-up was concluded in June 2024, with 1400 participants randomised (Fig. 1); the modified intention-to-treat population comprised 1389 participants (Table 1). Two (0.14%) participants died between randomisation and surgery, and in 17 (1.2%) participants, the planned surgical procedure was either cancelled or changed to a non-CABG/AVR procedure (Fig. 1). Both groups had similar EuroSCORE II, with the majority of participants (937; 67%) undergoing isolated CABG (Table 2).

**Table 1:** Baseline characteristics of the modified intention-to-treat population stratified by treatment allocation. Data are presented as median (IQR) or *n* (%). CCS, Canadian Cardiovascular Society; ICD, implantable cardiac defibrillator; IQR, interquartile range; NT-proBNP, N-terminal pro-brain natriuretic peptide; NYHA, New York Heart Association.

	Fio_2_ 50%	Fio_2_ 100%
	(*n*=695)	(*n*=694)
Age (yr), mean (range)	66 (29–85)	67 (30–85)
Female sex	112 (16)	121 (17)
Caucasian	679 (98)	679 (99)
Body mass index (kg m^−2^)	27.1 (25–31)	27 (25–30)
Comorbidities and performance		
Smoking, current or previous	453 (66)	438 (63)
Alcohol, excessive consumption	8 (1.2)	6 (0.9)
Heart failure	115 (17)	110 (16)
Hypertension	443 (64)	455 (66)
Type 2 diabetes mellitus	105 (15)	101 (15)
Type 1 diabetes mellitus	11 (1.6)	10 (1.5)
Pulmonary disease	72 (11)	81 (12)
Ischaemic heart disease	537 (77)	542 (78)
Previous myocardial infarction	122 (18)	141 (21)
Previous percutaneous coronary intervention	95 (14)	100 (15)
Previous coronary artery bypass grafting	5 (0.7)	6 (0.9)
Previous valve surgery	9 (1.3)	9 (1.3)
Previous stroke	55 (8.0)	69 (10.0)
Pacemaker or ICD	13 (1.9)	19 (2.8)
Dialysis	14 (2.0)	9 (1.3)
Frailty score ≥4	163 (33)	191 (39)
NYHA class ≥3	131 (25)	140 (27)
CCS class ≥3	67 (13)	63 (12)
NT-proBNP (pmol L^−1^)	35 (13–95)	37 (15–103)
Creatinine (μmol L^−1^)	85 (75–98)	85 (75–99)
Medications		
Acetylsalicylic acid	432 (63)	452 (66)
Non-vitamin K oral anticoagulants	59 (8.5)	57 (8.3)
Angiotensin converting enzyme inhibitor	175 (25)	175 (25)
Angiotensin receptor blocker	179 (26)	184 (27)
Statins	494 (72)	506 (74)
Beta blockers	302 (44)	316 (46)
Calcium blockers	187 (27)	183 (27)
Thiazides	111 (16)	110 (16)
Insulin	26 (3.8)	40 (5.8)

**Table 2:** Surgical characteristics of the modified intention-to-treat population stratified by treatment allocation. Data are presented as median (IQR) or *n* (%). AVR, aortic valve replacement; CABG, coronary artery bypass grafting; CPB, cardiopulmonary bypass; IQR, interquartile range.

	Fio_2_ 50%	Fio_2_ 100%
	(*n*=695)	(*n*=694)
Primary indication for surgery		
Coronary artery disease	367 (70)	351 (68)
Aortic stenosis	160 (30)	167 (32)
Performed surgery		
CABG	476 (69)	461 (67)
AVR	155 (23)	149 (22)
CABG+AVR	56 (8.1)	73 (11)
For CABG: no. of diseased coronary vessels		
1	33 (6.5)	45 (9.0)
2	102 (20)	107 (22)
3	249 (49)	231 (46)
For AVR: prosthesis type		
Biological	144 (22)	166 (25)
Mechanical	52 (7.8)	51 (7.7)
For AVR: echocardiographic gradients		
Aortic valve area (cm^2^)	0.8 (0.6–0.9)	0.7 (0.7–0.9)
Peak gradient (mm Hg)	72 (55–91)	65 (53–84)
Mean gradient (mm Hg)	45 (35–60)	40.8 (30–55)
Additional surgery		
Ascending aorta	9 (1.4)	4 (0.6)
Mitral valve	3 (0.5)	3 (0.5)
Tricuspid valve	0 (0.0)	2 (0.3)
Patent foramen ovale closure	0	1 (0.2)
Left atrial appendage closure	0	1 (0.2)
Duration of		
CPB (min)	88 (70–112)	87 (70–119)
Aortic cross clamp (min)	56 (41–76)	56 (40–80)
Reperfusion (min)	23 (17–29)	23 (17–30)

### Effect of intervention: arterial oxygen partial pressure levels during cardiopulmonary bypass

**Fig 2:**
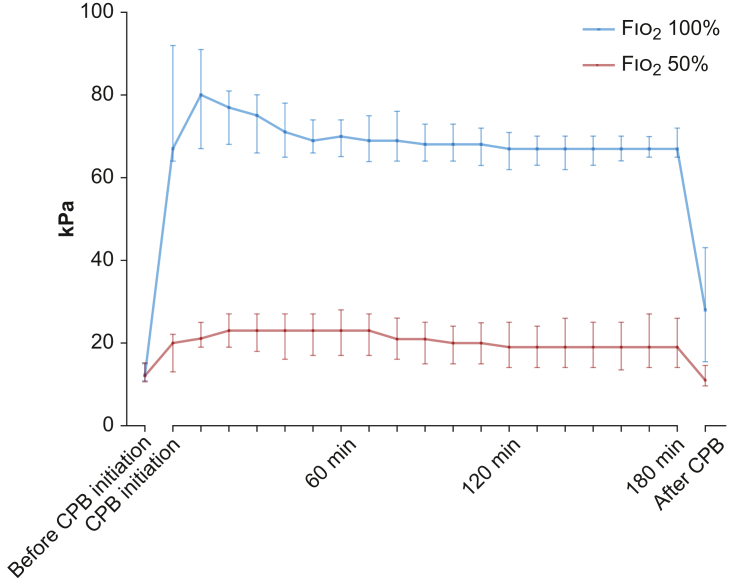
Median Pao_2_ levels with interquartile range during the intervention. Pao_2_ levels measured before initiation of cardiopulmonary bypass (CPB) and 10 min after conclusion of CPB. Furthermore, for each patient, the average Pao_2_ level over a 10-min period was recorded from initiation of CPB until 180 min after CPB. The median Pao_2_ level with interquartile range per 10-min interval from initiation of CPB is depicted.

Before initiation of CPB (i.e. before initiation of the oxygenation intervention), the median Pao_2_ level was 12 (IQR, 11–15) kPa in the restrictive oxygenation group, compared with 12 (IQR, 11–15) kPa in the liberal oxygenation group (Fig. 2). In the first 3 h after initiation of CPB, median Pao_2_ levels were between 19 and 23 kPa in the restrictive oxygenation group, compared with levels above 60 kPa in the liberal oxygenation group (Fig. 2). Ten minutes after weaning from CPB (i.e. still during the intervention period), the Pao_2_ level was 11 (IQR, 9.6–15) kPa in the restrictive oxygenation group and 28 (IQR, 15–43) kPa in the liberal oxygenation group (Fig. 2). A total of 69 (10%) patients in the restrictive oxygenation group received an Fio_2_ higher than 50% at any time point during the intervention period. The most common reason for increasing Fio_2_ above 50% was arterial oxygen saturation below 92% (*n*=58, 8.8%). In the liberal oxygenation group, 18 (2.7%) patients received an Fio_2_ lower than 100% at any time point during the intervention period.

### Primary composite outcome

**Fig 3:**
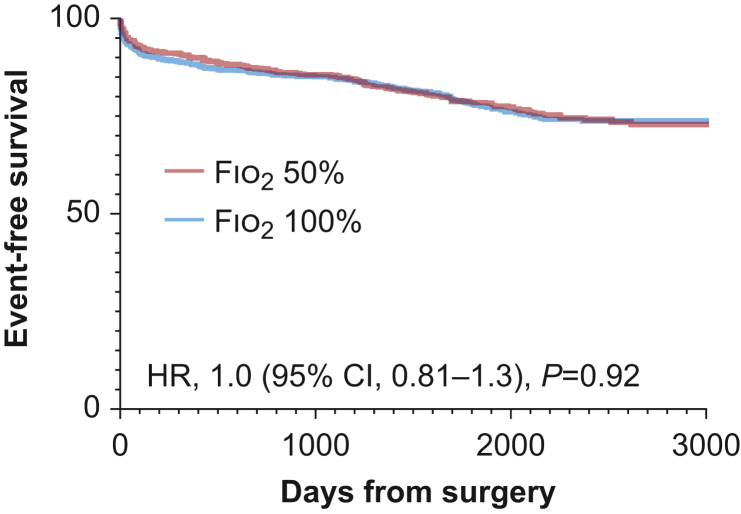
Time to first endpoint (death, renal failure requiring dialysis, stroke, new-onset/worsening heart failure) stratified by treatment allocation. Hazard ratios (HRs) with 95% confidence intervals (95% CIs) are presented for Fio_2_ 50% *vs* Fio_2_ 100%.

During a median follow-up period of 5.9 (IQR, 2.5–8.3) yr, 167/695 (24%) participants in the restrictive oxygenation group and 168 (24%) participants in the liberal oxygenation group met the primary endpoint (HR, 1.01; 95% CI, 0.81–1.3; *P*=0.92; Fig. 3). After adjustment for potential confounding factors, we found no differences in time to the first occurring primary endpoint between the restrictive and the liberal oxygenation allocation (HR, 1.1; 95% CI, 0.85–1.4). There was no significant interaction between the oxygen and the GLP-1 intervention (*P*_interaction_=0.40).

### Secondary analyses

The time to extubation and discharge from ICU and hospital (median length of stay, 6 days [IQR, 5–7 days]) were not different between participants receiving restrictive oxygenation and participants receiving liberal oxygenation (Supplementary material 2). Mortality in the restrictive *vs* the liberal oxygenation group was six participants (0.86%) *vs* 14 participants (2.0%) 30 days after surgery. At 1 yr, the mortality in the restrictive *vs* the liberal oxygenation group was 17 participants (2.5%) *vs* 26 participants (3.8%). Throughout the follow-up period, 86 (12%) participants receiving restrictive oxygenation and 98 (14%) participants receiving liberal oxygenation died. There was no difference in time to death between the two randomisation groups (HR, 0.89 [95% CI, 0.66–1.2]; Supplementary material 3 and 4). Additionally, there were no differences in time to renal failure, stroke, or heart failure taking competing risk of death into account (Supplementary material 5).

### Adverse events

There were no differences in the frequency of predefined adverse events between participants receiving restrictive oxygenation and participants receiving liberal oxygenation (Table 3).

**Table 3:** Predefined adverse events. Acute kidney injury defined according to KDIGO stage 2 or higher as doubling of S-creatinine or urine output below 0.5 ml kg^−1^ min^−1^ for over 12 h. Hypoglycaemia defined as blood glucose below 3 mmol L^−1^. Pancreatitis defined as serum amylase above three times upper normal limit. KDIGO, Kidney Disease: Improving Global Outcomes; LVEF, left ventricular ejection fraction. ∗Death was assessed during the follow-up period. Re-admission for cardiovascular causes was assessed for 12 months from index surgery. Other events assessed during index admission.

	Fio_2_ 50%	Fio_2_ 100%	*P*
	(*n*=695)	(*n*=694)	
Death∗	86 (12)	98 (14)	0.34
Surgical site infection	22 (3.2)	11 (1.6)	0.05
Acute kidney injury	40 (5.8)	30 (4.3)	0.22
Hypoglycaemia within 12 h	1 (0.14)	1 (0.14)	0.99
Pancreatitis	1 (0.14)	2 (0.29)	0.99
Reduction of LVEF >50% from baseline	11 (1.6)	7 (1.0)	0.34
Reoperation for bleeding	30 (4.3)	36 (5.2)	0.45
Reoperation for any cause	18 (2.6)	19 (2.7)	0.86
Post-surgery myocardial infarction	7 (1.0)	7 (1.0)	0.99
Re-admission for cardiovascular causes∗	170 (24)	193 (28)	0.16

### Sensitivity analyses

In the pre-planned sensitivity analyses, no differences were found in time to the composite endpoint within 180 days from surgery (Supplementary material 4 and 5). We found no significant effects of restrictive *vs* liberal oxygenation across subgroups (Supplementary material 6). For the primary endpoint analysis, we found no significant interaction between treatment allocation and EuroSCORE II (*P*_*interaction*_=0.87).

## Discussion

In this RCT, we found no significant difference in a composite endpoint comprising death, stroke, renal failure requiring dialysis, or new-onset worsening/worsening heart failure between restrictive and liberal oxygenation strategies during CPB and subsequent weaning. To our knowledge, this trial is the largest RCT to investigate oxygenation targets in CPB-assisted cardiac surgery.

The balance between ensuring adequate oxygen delivery and avoiding the potential harms of hyperoxia during anaesthesia has been debated extensively. Advocates of liberal oxygenation cite beneficial effects on surgical site infection rates, postoperative nausea and vomiting, and an extended safety margin in emergency scenarios. In contrast, concerns have been raised about hyperoxia-induced oxidative stress, ischaemia-reperfusion injury, cardiovascular dysfunction, increased risk of atelectasis, and possible vasoconstriction with impaired tissue perfusion and organ injury.^7^, ^8^, ^9^, ^10^, ^11^, ^12^^,^^21^^,^^22^ Despite the supraphysiological oxygen levels achieved in the present trial, we observed no evidence of harm, adding to a growing body of evidence suggesting that high intraoperative oxygen levels may be applied safely.

Previous clinical trials in this domain have been limited by small sample sizes and a significant risk of bias.^12^ Of 12 trials published between 1991 and 2016, only one enrolled more than 100 patients.^23^ That trial randomised 298 patients undergoing CPB-assisted cardiac surgery to a Pao_2_ of 10–12 kPa (maintaining Sao_2_ above 97%) *vs* Fio_2_ titrated to an Sao_2_ of more than or equal to 99%. In that trial, no significant differences were found in acute kidney injury, biomarkers of organ dysfunction, length of stay, or mortality, albeit the trial was likely underpowered for the latter outcomes.^23^

Three trials investigating intraoperative oxygenation were conducted simultaneously with our trial.^24^, ^25^, ^26^ One trial randomised 100 patients undergoing CABG to an Fio_2_ of 100% *vs* an Fio_2_ of minimum 35% to maintain a Pao_2_ between 13 and 20 kPa during CPB, finding no significant differences in cognitive outcomes up to 6 months after operation.^24^ Another single-centre trial randomised 200 patients undergoing elective cardiac surgery to an Fio_2_ of 100% *vs* an Fio_2_ targeted to an Sao_2_ of 95–97%.^26^ Although increased oxidative stress was noted in the hyperoxic group, no differences in postoperative kidney injury or markers of organ injury were found.^26^ A third trial randomised 330 patients to an Fio_2_ of 100% *vs* an Fio_2_ targeted at achieving a Pao_2_ of less than 13 kPa during CPB,^25^ and similarly found no significant differences in postoperative atrial or ventricular arrhythmias, major cardiovascular events, or length of stay.^25^ As such, our findings support a growing body of clinical evidence indicating no clear benefit or harm of liberal oxygenation in terms of major patient-centred outcomes during cardiac surgery involving CPB.

We also found no significant difference in surgical site infection rates between the two oxygenation strategies. Although this observation may be limited by the low rates of surgical site infection in the trial, it is consistent with a recent systematic review and meta-analysis suggesting no significant association between high inspired oxygen fractions (Fio_2_ 80% *vs* 30%) and surgical site infection rates, mortality, or length of stay in noncardiac surgery populations.^27^

The trial results should be interpreted in light of the following limitations. The trial was conducted at a single Nordic centre. It included mainly male patients and only elective and urgent cases, which may limit generalisability. Notably, the effects of oxygen targets in higher-risk populations undergoing CPB-assisted heart surgery remain unknown. The ‘restrictive’ group, despite receiving an Fio_2_ of 50%, attained Pao_2_ levels of 19–23 kPa, highlighting a potential to explore even lower oxygen targets in future trials. Furthermore, 10% of patients in the ‘restrictive’ group received higher oxygen concentrations to achieve Sao_2_ levels of at least 92%, and the results should be interpreted in light of this safety mechanism. The trial was powered to detect an absolute risk reduction of 5.7%. As such, smaller effect sizes of the intervention on the primary endpoint cannot be ruled out. Furthermore, other effects on organ function, such as pulmonary function/atelectasis, and markers of cerebral, cardiac, and renal injury were not explored and could be a focus of future trials. The trial event rate was lower than anticipated, extending the required follow-up period; however, sensitivity analyses did not suggest a differential treatment effect after 180 days. In patients undergoing abdominal surgery, a previous trial has suggested a significant detrimental effect of an Fio_2_ of 80% *vs* 30% on survival (HR, 1.30 [1.03–1.64]) out to over 1000 days, suggesting that a long follow-up period may be needed to detect differences in outcome^28^; however, whether perioperative oxygenation targets can impact long-term outcomes remains speculative to some extent. When designing the trial, we assumed that there would be no interaction between the two study treatments in the factorial design. Although the interaction term was non-significant (*P*=0.40), we cannot rule out that a smaller interaction could have affected the risk of type II error. Finally, although the trial was blinded for participants, investigators, and endpoint assessors, blinding of surgical and anaesthesia staff was not feasible. Considering the objective definitions of the primary endpoint, the long follow-up, and blinding of endpoint assessors, we consider the likely impact to be minimal.

In summary, no differences were observed in mortality, dialysis-dependent renal failure, stroke, or new-onset or worsening heart failure between a restrictive oxygenation strategy (Fio_2_ 50%) and a liberal oxygenation strategy (Fio_2_ 100%) during CPB for CABG, AVR, or both.

## Authors’ contributions

Conception and design: SW, CHM, JK, HMS, PSO, DEH, JR, HR, SB, CH, LK, JCN

Acquisition of data: SW, JK, ADM, JBK, JCN

Analysis of data: SW, JK, ADM, JBK, JCN

Drafting the manuscript: SW

Critically revising the manuscript: CHM, JK, ADM, HMS, JBK, PSO, DEH, JR, HR, SB, CH, LK, JCN

Interpretation of data: all authors

Final approval: all authors

Agreed to be accountable for all aspects of the work: all authors

## Funding

Læge Sofus Carl Emil Friis og Hustru Olga Doris Friis' Legat (2017; Danish Krone (DKK) 461,180); Aase og Ejnar Danielsens Fond 2017 (grant no. 10-001976; DKK 200,000); Danish Heart Foundation (2019, grant no. 19-R133-A9174-22144; DKK 1,000,000); The Heart Centre Research Foundation (2017; DKK 160,000); Lundbeck Foundation (R186-2015-2132).

## Declarations of interest

LK has received speaker honoraria from Astra Zeneca, Boehringer, Novartis, and Novo Nordisk. The other authors declare that they have no conflicts of interest.

## References

[1] Caliskan E., Misfeld M., Sandner S. (2022). Clinical event rate in patients with and without left main disease undergoing isolated coronary artery bypass grafting: results from the European DuraGraft Registry. Eur J Cardiothorac Surg.

[2] Modolo R., Chichareon P., Kogame N. (2019). Contemporary outcomes following coronary artery bypass graft surgery for left main disease. J Am Coll Cardiol.

[3] Houlind K., Kjeldsen B.J., Madsen S.N. (2012). On-pump versus off-pump coronary artery bypass surgery in elderly patients results from the Danish On-Pump Versus Off-Pump Randomization Study. Circulation.

[4] Rosner M.H., Okusa M.D. (2006). Acute kidney injury associated with cardiac surgery. Clin J Am Soc Nephrol.

[5] Bellomo R., Auriemma S., Fabbri A. (2008). The pathophysiology of cardiac surgery-associated acute kidney injury (CSA-AKI). Int J Artif Organs.

[6] Qadan M., Akça O., Mahid S.S., Hornung C.A., Polk Jr HC. (2009). Perioperative supplemental oxygen therapy and surgical site infection. A meta-analysis of randomized controlled trials. Arch Surg.

[7] Topcu A.C., Bolukcu A., Ozeren K., Kavasoglu T., Kayacioglu I. (2021). Normoxic management of cardiopulmonary bypass reduces myocardial oxidative stress in adult patients undergoing coronary artery bypass graft surgery. Perfusion.

[8] Inoue T., Ku K., Kaneda T., Zang Z., Otaki M., Oku H. (2002). Cardioprotective effects of lowering oxygen tension after aortic unclamping on cardiopulmonary bypass during coronary artery bypass grafting. Circulation.

[9] Spoelstra-De Man A.M.E., Smit B., Oudemans-Van Straaten H.M., Smulders Y.M. (2015). Cardiovascular effects of hyperoxia during and after cardiac surgery. Anaesthesia.

[10] Belboul A., Al-khaja N., Ericson C. (1991). The effect of hyperoxia during cardiopulmonary bypass on blood cell rheology and postoperative morbidity associated with cardiac surgery. J Extra Corpor Technol.

[11] Joachimsson P.-O., Sjöberg F., Forsman M., Johansson M., Casimir Ahn H., Rutberg H. (1996). Adverse effects of hyperoxemia during cardiopulmonary bypass. J Thorax Cardiovasc Surg.

[12] Heinrichs J., Lodewyks C., Neilson C., Abou-Setta A., Grocott H.P. (2018). The impact of hyperoxia on outcomes after cardiac surgery: a systematic review and narrative synthesis. Can J Anesth.

[13] Young R.W. (2012). Hyperoxia: a review of the risks and benefits in adult cardiac surgery. J Extra Corpor Technol.

[14] Roberts S.M., Cios T.J. (2019). Con: hyperoxia should not be used routinely in the management of cardiopulmonary bypass. J Cardiothorac Vasc Anesth.

[15] Heinrichs J., Grocott H.P. (2019). Pro: hyperoxia should be used during cardiac surgery. J Cardiothorac Vasc Anesth.

[16] Wahba A., Milojevic M., Boer C. (2020). EACTS/EACTA/EBCP guidelines on cardiopulmonary bypass in adult cardiac surgery. Eur J Cardiothorac Surg.

[17] Wiberg S., Kjaergaard J., Møgelvang R. (2021). Efficacy of a glucagon-like peptide-1 agonist and restrictive versus liberal oxygen supply in patients undergoing coronary artery bypass grafting or aortic valve replacement: study protocol for a 2-by-2 factorial designed, randomised clinical trial. BMJ Open.

[18] Kellum J.A., Lameire N., Aspelin P. (2012). Kidney Disease: Improving Global Outcomes (KDIGO) Acute Kidney Injury Work Group. KDIGO clinical practice guideline for acute kidney injury. Kidney Int Suppl.

[19] Thygesen K., Alpert J.S., Jaffe A.S. (2012). Third universal definition of myocardial infarction. Eur Heart J.

[20] Nashef S.A.M., Roques F., Sharples L.D. (2012). Euroscore II. Eur J Cardiothorac Surg.

[21] Weenink R.P., de Jonge S.W., van Hulst R.A. (2020). Perioperative hyperoxyphobia: justified or not? Benefits and harms of hyperoxia during surgery. J Clin Med.

[22] Pizov R., Weiss Y.G., Oppenheim-Eden A. (2000). High oxygen concentration exacerbates cardiopulmonary bypass-induced lung injury. J Cardiothorac Vasc Anesth.

[23] McGuinness S.P., Parke R.L., Drummond K., Willcox T., Bailey M. (2016). A multicenter, randomized, controlled phase IIb trial of avoidance of hyperoxemia during cardiopulmonary bypass. Anesthesiology.

[24] Shaefi S., Shankar P., Mueller A.L. (2021). Intraoperative oxygen concentration and neurocognition after cardiac surgery a randomized clinical trial. Anesthesiology.

[25] Abou-Arab O., Huette P., Martineau L. (2019). Hyperoxia during cardiopulmonary bypass does not decrease cardiovascular complications following cardiac surgery: the CARDIOX randomized clinical trial. Intensive Care Med.

[26] Lopez M.G., Shotwell M.S., Hennessy C. (2024). Intraoperative oxygen treatment, oxidative stress, and organ injury following cardiac surgery: a randomized clinical trial. JAMA Surg.

[27] Høybye M., Lind P.C., Holmberg M.J. (2022). Fraction of inspired oxygen during general anesthesia for non-cardiac surgery: systematic review and meta-analysis. Acta Anaesthesiol Scand.

[28] Meyhoff C.S., Jorgensen L.N., Wetterslev J., Christensen K.B., Rasmussen L.S. (2012). Increased long-term mortality after a high perioperative inspiratory oxygen fraction during abdominal surgery: follow-up of a randomized clinical trial. Anesth Analg.

