# Supplementary material for: Restrictive versus liberal oxygenation in patients undergoing cardiopulmonary bypass-assisted heart surgery: a randomised controlled trial
Source: Br J Anaesth. 2025 Aug 19;135(6):1618–25. doi: 10.1016/j.bja.2025.08.005 (PMC12799424; doi:10.1016/j.bja.2025.08.005)
Supplement: Multimedia component 1 [file mmc1.docx]

Restrictive versus liberal oxygenation in patients undergoing cardiopulmonary bypass-assisted heart surgery: randomised controlled trial.

Sebastian Wiberg^,,^, Christian H Møller, Jesper Kjaergaard^,^, Astrid D Mikkelsen, Hasse-Møller Sørensen, Joakim B Kunkel, Peter S Olsen, Dan E Høfsten, Jesper Ravn, Hanne Ravn, Søren Boesgaard, Christian Hassager^,^, Lars Køber, Jens C Nilsson

**Supplementary Material**

Contents

[Supplementary Data 1. Eligibility criteria 2](#_Toc206087538)

[Supplmentary data 2- secondary endpoints. 3](#_Toc206087539)

[Extubation 3](#_Toc206087540)

[ICU discharge 3](#_Toc206087541)

[Hospital discharge 3](#_Toc206087542)

[Supplementary Data 3- composite endpoints 4](#_Toc206087543)

[Supplementary Data 4- time to first endpoint. 5](#_Toc206087544)

[Supplementary Data 5- Time to the individual components of the composite endpoint 6](#_Toc206087545)

[Supplementary Data 6- subgroup analyses. 7](#_Toc206087546)

[Supplementary Data 7- Acknowledgements 8](#_Toc206087547)

# Supplementary Data 1. Eligibility criteria

| **Inclusion criteria** |
| --- |
| - Informed written consent - Age ≥ 18 years - CABG and/or AVR, irrespective of other concomitant valve surgery |
|  |
| **Exclusion criteria** |
| - Active treatment with GLP-1 analogs - Obstructive hypertrophic cardiomyopathy, active myocarditis, constrictive pericarditis - Hyperthyroidism or untreated hypothyroidism - History of, or active pancreatitis - Emergency surgery; urgent surgery (i.e. the following days) are eligible - Known allergy towards exenatide/Byetta or albumin (vehicle) - On the urgent waiting list for a heart transplant (UNOS category 1A or 1B or equivalent) - Recipient of any major organ transplant (e.g. Heart, lung, liver) - Receiving of has received cytotoxic or cytostatic chemotherapy and/or radiation therapy for treatment of malignancy within 6 months before randomization - Clinical evidence of current malignancy, with the exceptions of: basal or squamous cell carcinoma, cervical intraepithelial neoplasia, prostate cancer with a life expectancy of > 2.5 years - Currently enrolled in, or within 30 days from ending participation in other investigational drug trials for the treatment of diabetes or malignant obesity. Participation in other non-pharmacological trials is not an exclusion criterion - Recent, within 3 months, history of alcohol or drug abuse disorder, based on self-report - Pregnancy or currently breast feeding - Any condition or situation that, in the investigator’s opinion, could put the subject at significant risk, confound the trial’s results or interfere with the subject’s participation in the trial (specific reasons will be provided) - Previous participation in the GLORIOUS trial |
|  |

GLP-1: glucagon-like peptide; UNOS: United network for organ sharing

# Supplmentary data 2- secondary endpoints.

## Extubation

Patients receiving restrictive oxygenation were extubated a median of 9.0 (IQR 7.7 to 12) hours from intubation, and patients receiving liberal oxygenation were extubated a median of 9.3 (IQR 7.7 to 12) hours from intubation.

## ICU discharge

Patients receiving restrictive oxygenation were discharged from the ICU after a median of 21 (IQR 18-23) hours, and patients receiving liberal oxygenation were discharged from the ICU after a median of 21 (IQR 19-23) hours.

## Hospital discharge

Patients receiving restrictive oxygenation were discharged from hospital after a median length of stay of 6 (IQR 5 to 7) days, and patients receiving liberal oxygenation were discharged from hospital after a median length of stay of 6 (IQR 5 to 7) days.

# Supplementary Data 3- composite endpoints

Time to the individual components of the composite endpoint (death, renal failure requiring dialysis, stroke, new onset/worsening heart failure) stratified by treatment allocation

**
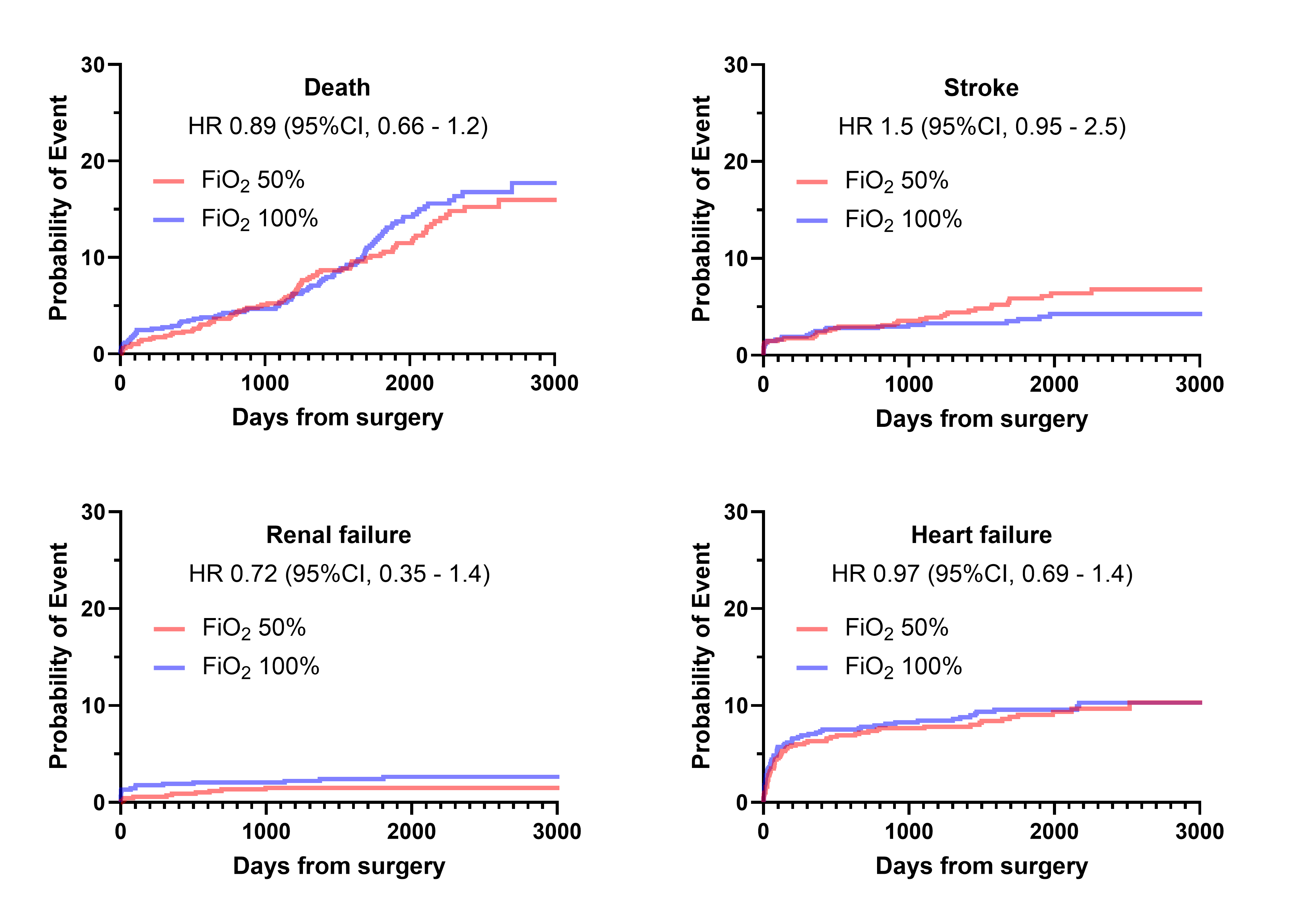
**

Models of stroke, renal failure, and now onset or worsening heart failure take competing risk of death into account. Hazard ratios with 95% confidence intervals are presented for FiO_2_ 50% versus FiO_2_ 100%.

Stroke is defined as clinical stroke as diagnosed by the clinical physician.

Renal failure is defined renal failure requiring renal replacement therapy.

Heart failure is defined as need for mechanical circulatory support at the ICU, inability to close the sternum due to hemodynamic instability and/or need for inotropic hemodynamic support more than 48 hours after initiation of the first surgical procedure after randomization. In addition, any admission for heart failure during follow-up after discharge from the index admission.

# Supplementary Data 4- time to first endpoint.

Time to first endpoint (death, renal failure requiring dialysis, stroke, new onset/worsening heart failure) within 180 days stratified by treatment allocation


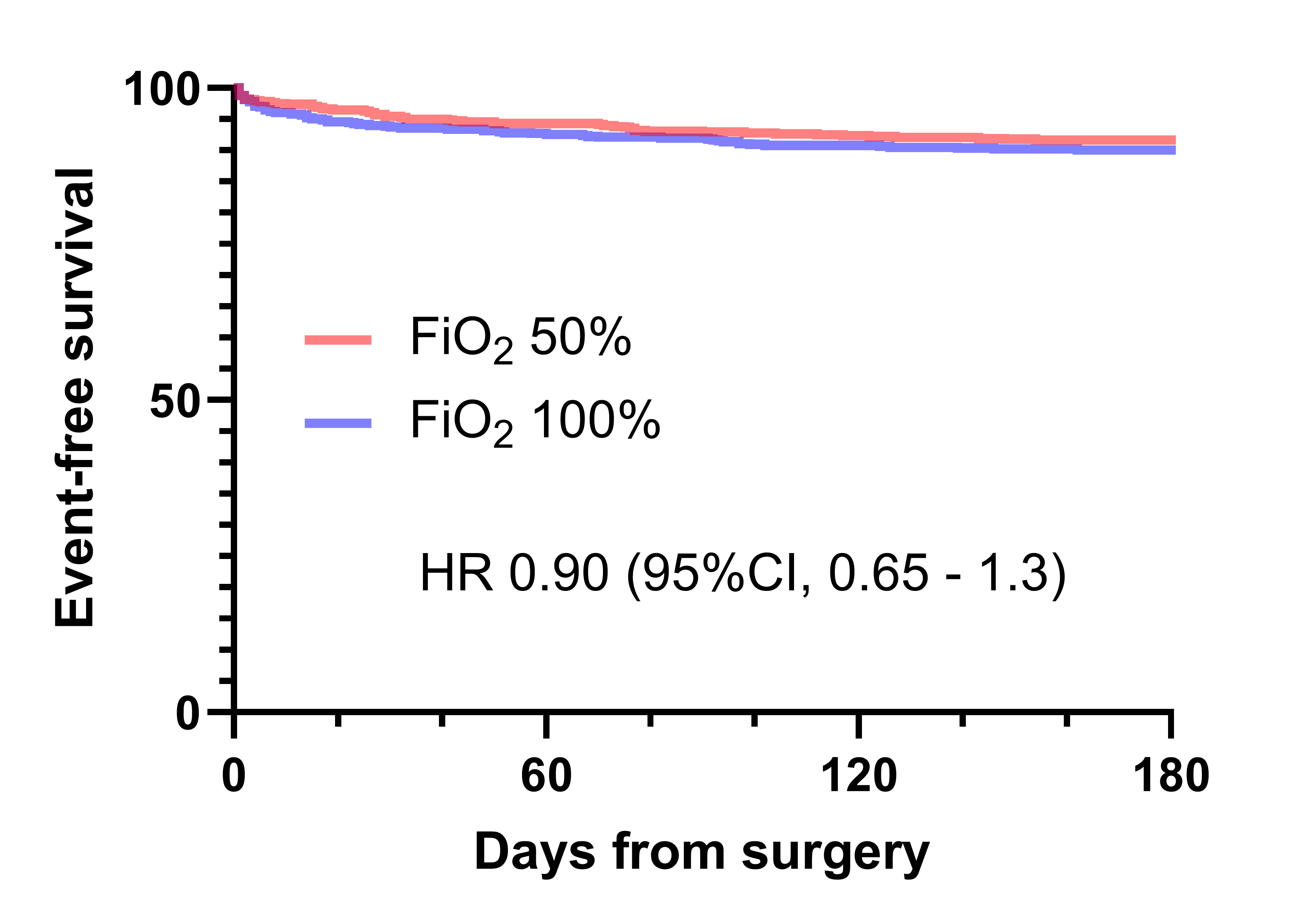


Hazard ratios with 95% confidence intervals are presented for FiO_2_ 50% versus FiO_2_ 100%.

# Supplementary Data 5- Time to the individual components of the composite endpoint

Time to the individual components of the composite endpoint (death, renal failure requiring dialysis, stroke, new onset/worsening heart failure) within 180 days stratified by treatment allocation


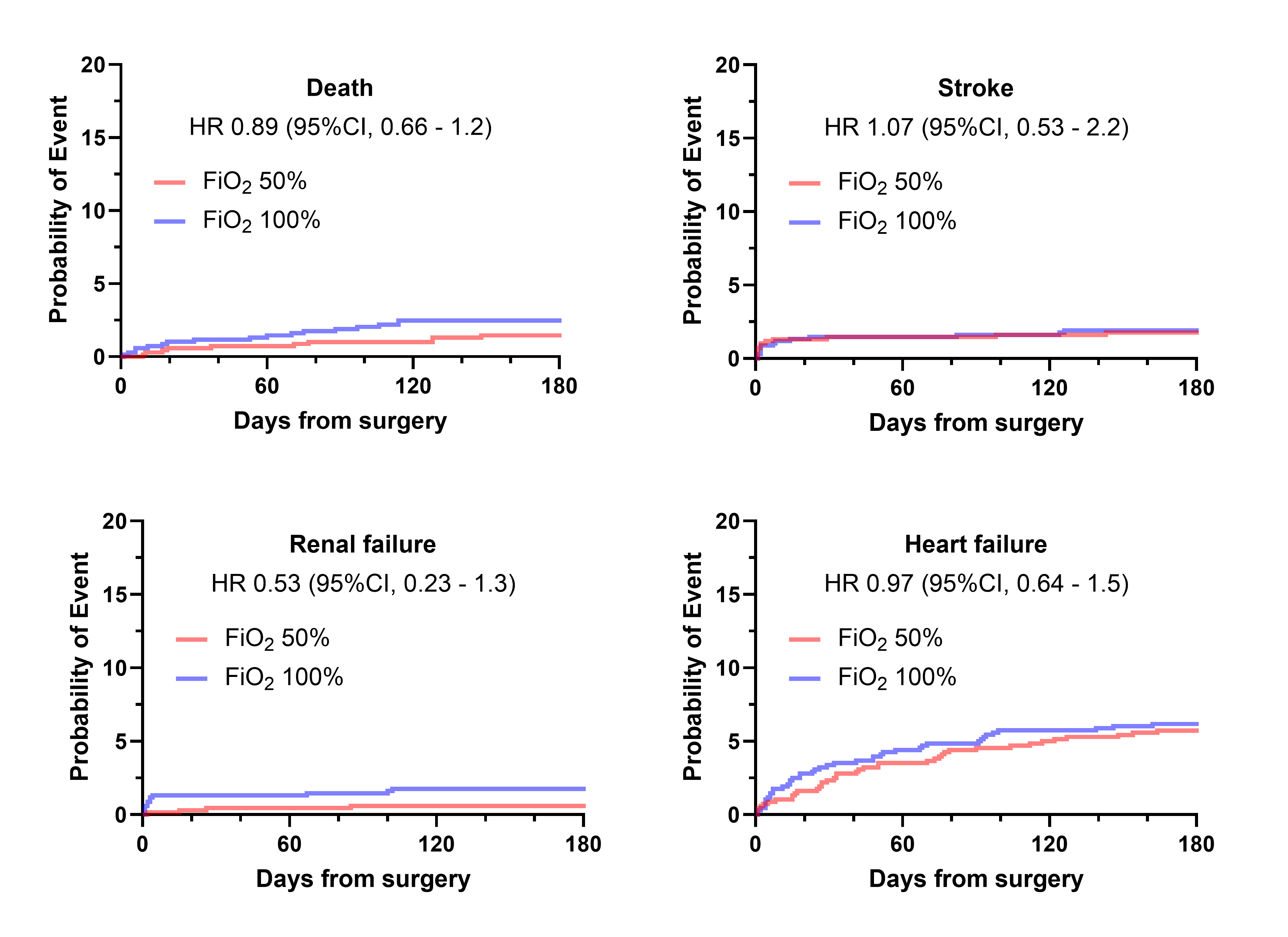


Models of stroke, renal failure, and now onset or worsening heart failure take competing risk of death into account. Hazard ratios with 95% confidence intervals are presented for FiO_2_ 50% versus FiO_2_ 100%.

Stroke is defined as clinical stroke as diagnosed by the clinical physician.

Renal failure is defined as renal failure requiring renal replacement therapy.

Heart failure is defined as need for mechanical circulatory support at the ICU, inability to close the sternum due to hemodynamic instability and/or need for inotropic hemodynamic support more than 48 hours after initiation of the first surgical procedure after randomization. In addition, any admission for heart failure during follow-up after discharge from the index admission.

# Supplementary Data 6- subgroup analyses.

Effects of restrictive versus liberal oxygenation in selected sub-groups

**
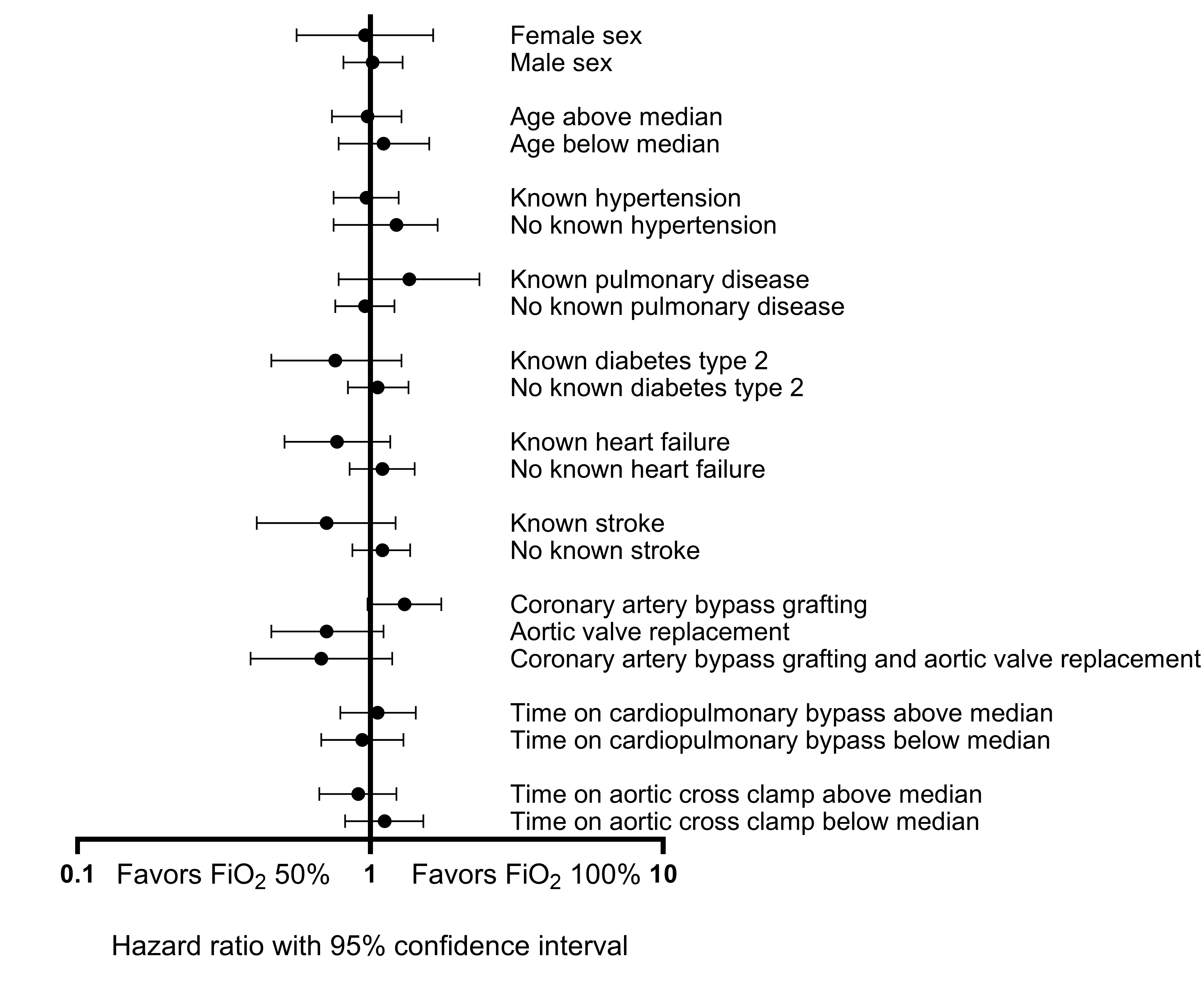
**

# Supplementary Data 7- Acknowledgements

The trial steering committee would like to extent our sincere gratitude to the clinical staff at Department of Cardiothoracic Anesthesiology and Intensive care including the post-surgical fast-track unit, Department of Cardiothoracic Surgery, and Cardiac Intensive Care Unit 2143. Your dedication remains crucial for the conduct of clinical trials in the Heart Centre. Furthermore, we would like to thank Clinical Research Unit (Klinisk Forskningsenhed) for their aid in the day-to-day conduct of the trial.
